# Gamma-glutamyl transferase variability can predict the development of end-stage of renal disease: a nationwide population-based study

**DOI:** 10.1038/s41598-020-68603-0

**Published:** 2020-07-15

**Authors:** Da Young Lee, Kyungdo Han, Ji Hee Yu, Sanghyun Park, Jee-In Heo, Ji A. Seo, Nam Hoon Kim, Hye Jin Yoo, Sin Gon Kim, Seon Mee Kim, Kyung Mook Choi, Sei Hyun Baik, Yong Gyu Park, Nan Hee Kim

**Affiliations:** 1 Division of Endocrinology and Metabolism, Department of Internal Medicine, Korea University College of Medicine, Seoul, Republic of Korea; 2 Department of Biostatics, College of Medicine, The Catholic University of Korea, 222, Banpo-daero, Seocho-gu, Seoul, 06591 Republic of Korea; 3 Department of Family Medicine, Korea University College of Medicine, Seoul, Republic of Korea; 4 Division of Endocrinology and Metabolism, Department of Internal Medicine, Korea University Ansan Hospital, Korea University College of Medicine, 123, Jeokgeum-ro, Danwon-gu, Ansan-si, Gyeonggi-do 15355 Republic of Korea

**Keywords:** Biomarkers, Endocrine system and metabolic diseases, Kidney diseases, Metabolic disorders

## Abstract

The aim of this study is to investigate whether GGT variability is able to predict the risk of end-stage renal disease (ESRD). The study subjects were Koreans who conducted health exams supported by the Korean National Health Insurance Corporation during 2009–2012 (baseline). After excluding individuals aged < 40 years, heavy alcoholics, or those with histories of chronic liver disease or ESRD, we followed 6,058,995 individuals. We calculated the average successive variability (ASV) of GGT values during the 5 years before the baseline as a parameter of variability. Using Cox proportional analyses, we evaluated the risk of ESRD according to GGT ASV quartiles, defined as the initiation of renal replacement therapy or kidney transplantation, or December 31, 2016. During 38,663,279.3 person-years of follow-up, 12,057 cases of ESRD were identified. Compared with GGT ASV quartile 1, the risk of ESRD was higher in ASV quartiles 3–4 and increased serially, even after adjustment for several metabolic parameters, baseline renal function, presence of comorbidities, low income, and baseline GGT and hemoglobin level. The fully adjusted hazard ratios (95% confidence intervals) of GGT ASV quartiles 3 and 4 were 1.06 (1.01–1.12) and 1.12 (1.06–1.18), respectively. In conclusion, GGT variability is a putative risk factor for ESRD in Koreans.

## Introduction

The number of patients who suffer from end-stage renal disease (ESRD) is increasing on a global scale^1^, and the estimated risk for ESRD in individuals without albuminuria is 1.1 in 10,000 patient-years^2^. Given that ESRD imposes a large economic burden as well as morbidity and mortality^3^, it is valuable to identify the risk factors for ESRD later in life.

Several anthropometric and metabolic factors, such as age, being male, obesity, diabetes, hypertension, and dyslipidemia have been demonstrated as risk factors for chronic kidney disease (CKD) in the previous studies^4,5^. Beyond those, serum levels of gamma-glutamyl transferase (GGT) have been suggested as a risk factor for CKD^6–10^ and ESRD^5,11^. Serum GGT has been used as an indicator of alcohol intake and liver dysfunction^12^. In addition, because GGT is a cell-surface enzyme that metabolizes extracellular glutathione, which is the main antioxidant in mammalian cells^13^, elevated serum GGT is often considered as an early and sensitive marker of oxidative stress^14^. Previous studies have shown that elevated GGT levels can predict diabetes, hypertension, metabolic syndrome, and CVD events that are risk factor for CKD^15^. However, in previous studies the significance of GGT as a predictor of renal function varied by sex^5,8^.

Previous epidemiological studies of the single measurement of biological variables had the common weak point that they did not reflect changes over time. Therefore, the intraindividual variability of physiological measures is now the focus of much research attention as a risk factor for health-related outcomes^16–19^. Recently, as a predictive marker for ESRD, Kim et al. proposed variability in total cholesterol (TC) levels^17^. In the coefficient of variation (CV) of TC, the highest quartile group showed more than twice the risk of ESRD compared with the lowest quartile group.

Only one previous study has calculated variability in serum GGT. The National Health and Nutrition Examination Survey III examinations found high short-term intraindividual variability within 17.5-day intervals^20^, but the clinical implications of that variability have not yet been studied. Apart from the risk of elevated GGT, fluctuation in GGT levels could present an additional risk. Therefore, we here use nationally representative data from the National Health Insurance System (NHIC) in Korea to investigate whether variability in GGT has a predictive effect on the risk of ESRD.

## Methods

### Selection of study subjects

We used the medical records provided by the Korean NHIC, which covers ~ 97% of the Koreans. The NHIC managed by the government of Korea is the single health insurance system and has the health information, including an eligibility data (e.g., age, sex, income level, and socioeconomic information), health check-up database (general health exams and questionnaires regarding lifestyle), medical treatment database (based on the medical bills submitted by health service providers), and medical care institution database^21,22^. Individuals included in the NHIC are recommended to do a standardized health check-up every 1 to 2 years. The entire database is now open to approved researchers.

**Figure 1:**
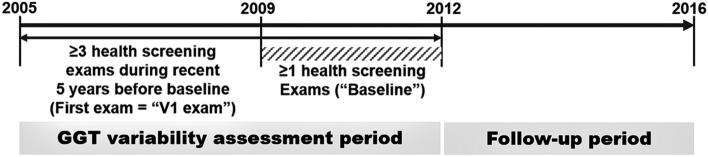
Selection of the study subjects. GGT, gamma-glutamyl transferase.

We chose individuals who had undergone health examinations more than once between January 1, 2009, and December 31, 2012 (referred to baseline) and more than twice in the 5 years immediately before the baseline examination (Fig. 1). Among them, we excluded those aged younger than 40 years and those with previous histories of liver cirrhosis, chronic hepatitis, liver malignancy, ESRD, or drinking ≥ 30 g/day of alcohol (estimated by self-reported questionnaires used in the baseline health examination), along with those who were missing data in those inclusion criteria. After those exclusions, 6,058,995 individuals (2,977,518 men and 3,081,477 women) were study subjects of present study (Supplementary Fig. S1).

The protocol of this study was approved by the Institutional Review Board of the Korea University Ansan Hospital (Institutional Review Board number 2018AS0161) and the official review committee in the NHIC and was carried out in accordance with the Helsinki Declaration of 1975.

### Definition of GGT variability and GGT percent change

GGT variability was assessed using the average successive variability (ASV), standard deviation (SD), and CV of serial measurements of GGT during the 5 years before the baseline examination (GGT variability assessment period in Fig. 1). The GGT level during the baseline examination was also included in the calculation of GGT variability. The first health examination during the 5 years before the baseline is called the V1 examination.

GGT ASV was calculated using the following equation:

$$\text{GGT}\;\text{ASV} = \frac{\sum |{x}_{i+1}-{x}_{i}|}{n-1}$$

${x}_{i}$ = each GGT value, $n$ = number of GGT measurements (three to five times per subject).

### Study outcomes

The endpoints were the first diagnosis of ESRD, defined as the initiation of renal replacement therapy or kidney transplantation under International Classification of Disease, 10th Revision (ICD-10) codes N18-19, Z49, Z90, Z94, or Z99.2, and December 31, 2016^17^. All dialysis procedures are reimbursed under registration in Korea, so we could identify all cases of renal replacement therapy using the claim codes for hemodialysis (O7011-O7020 or V001), peritoneal dialysis (O7071-O7075 or V003), and kidney transplantation (R3280)^17^. We excluded acute renal failure cases, defined as individuals with transiently undertaken renal replacement therapy, including acute peritoneal dialysis or continuous renal replacement therapy without a previous history of CKD. Deceased cases, defied using the nationwide death certificate data from the Korea National Statistical Office, were regarded as dropouts and excluded from the analyses. The time interval between the last examination between 2009 and 2012 and incident ESRD or December 31, 2016, is called the follow-up period (Fig. 1).

### Anthropometric and laboratory measurements

During each visit, individuals completed a questionnaire addressing eligibility information, lifestyle habits, and medical history. Cigarette smoking was categorized as never, ex-smokers, and current smokers. Alcohol drinking status was stratified by near abstinence or moderate (alcohol < 30 g/day). Doing more than 20 min of vigorous- or more than 30 min of moderate-intensity exercise at least once per week was defined as regular exercise^23^.

Subjects whose body mass index (BMI) was more than including 25 kg/m^2^ were classified as obese, according to the revised Asia–Pacific criteria of obesity^24^. Waist circumference was estimated at the middle point between the iliac crest and the rib cage. Blood pressure (BP) was measured using a standardized sphygmomanometer after a 5-min rest.

A systolic BP ≥ 140 mmHg, diastolic BP ≥ 90 mmHg, or at least one claim per year for the prescription of antihypertensive medications under ICD-10 codes I10–I15 was stratified as the presence of hypertension. Low-income status was identified at the lowest 20%.

After overnight fasting of > 8 h, venous blood samples were collected in the morning. Serum levels of fasting glucose, lipid profiles, aspartate aminotransferase (AST), alanine aminotransferase (ALT), GGT, creatinine, and hemoglobin were measured. The estimated glomerular filtration rate (eGFR) using the Modification of Diet in Renal Disease formula was calculated^25^ and individuals whose eGFR was less than 60 mL/min/1.73 m^2^ were classified as CKD^26^. The presence of diabetes was defined as a fasting glucose level ≥ 7 mmol/L, or at least one prescription of anti-glycemic agents per year under ICD-10 codes E10–14 based on the claim database. Dyslipidemia was defined as TC levels ≥ 6**.**21 mmol/L or at least one prescription history of anti-hyperlipidemic agents per year under ICD-10 code E78. The individuals having nonalcoholic fatty liver disease (NAFLD) was identified using the fatty liver index (FLI) ≥ 60, which is a noninvasive method to identify NAFLD in epidemiologic studies, including the Asians^27–29^. The history of heart disease or stroke was determined by self-report. Admission episodes for heart failure or myocardial infarction were identified using claim data for hospital admissions.

In accordance with the Korean Association of Laboratory Quality Control, the quality control for the laboratory tests was conducted.

### Statistical analysis

We stratified the subjects according to their quartiles of baseline GGT and GGT ASV. According to each quartile and incident ESRD, we compared the baseline characteristics using *t*-tests or analysis of variance for continuous variables and chi-square tests for categorical variables. Data are presented as mean ± SD, geometric mean (95% confidence intervals [CIs]), or number (%). For analysis, ALT, AST, GGT, and triglyceride levels were log-transformed.

To estimate the risk for the development of ESRD, we conducted Cox proportional hazard analyses according to the quartiles of baseline GGT and GGT ASV, using each quartile 1 as the reference. We adjusted for confounders at baseline using three models. In Model 1, is age, sex, eGFR, and BMI were adjusted. Model 2 is the same as model 1 plus adjustments for moderate drinking, current smokers, regular exercise, and the presence of hypertension, diabetes, and dyslipidemia. Model 3 is further adjusted for hemoglobin and income in the lowest 20%. In the GGT variability analysis, we also adjusted for baseline GGT level. In addition, we examined the effects of GGT variability on ESRD according to the baseline GGT quartiles to show that our results are consistent regardless of the GGT level.

We performed subgroup analyses after dividing the subjects by age, sex, obesity, eGFR, anemia, current smokers, alcohol drinking, income status, presence of hypertension and diabetes. We obtained hazard ratios (HRs) and 95% CIs for ESRD in GGT ASV quartile 4 versus quartiles 1–3 using model 3 except for the variables that categorized each subgroup.

We did sensitivity analyses by excluding the subjects who developed ESRD within 1 year. We also repeated the same analyses using SD and CV to see whether they showed similar results. For the Cox proportional hazards analyses, we conducted the variable inflation factor for all covariates of less than 2.0, and we found that there was no relevant multicollinearity among covariates. For statistical analysis, SAS version 9.3 (SAS Institute Inc., Cary, NC, USA) was used. A *p*-value of < 0.05 was considered to be statistically significant.

## Results

Subjects in GGT ASV quartile 4 were older, more obese, and had higher BP and more comorbidities than those in quartile 1 (Table 1). When study subjects were stratified by baseline GGT quartile, the metabolic variables got worse as the baseline GGT quartile increased (Supplementary Table S1).

**Table 1:** Baseline characteristics of the study subjects according to the quartiles of gamma-glutamyl transferase variability assessed by average successive variability.

Characteristics	ASV Q1 (n = 1,515,267)	ASV Q2 (n = 1,514,102)	ASV Q3 (n = 1,514,878)	ASV Q4 (n = 1,514,748)	*P* value
**Age (years)**	55.0 ± 10.3	55.0 ± 10.2	55.4 ± 10.3	56.2 ± 10.4	< 0.001
40–64	1,237,366 (81.7)	1,242,095 (82.0)	1,230,212 (81.2)	1,196,312 (79.0)	
≥ 65	277,901 (18.3)	272,007 (18.0)	284,666 (18.8)	318,436 (21.0)	
Sex, male (%)	744,918 (49.2)	743,772 (49.1)	744,449 (49.1)	744,379 (49.1)	< 0.001
BMI (kg/m^2^)	23.7 ± 2.9	23.9 ± 2.9	24.0 ± 3.0	24.1 ± 3	< 0.001
WC (cm)	80.4 ± 8.4	80.8 ± 8.4	81.2 ± 8.4	81.6 ± 8.4	< 0.001
Systolic BP (mmHg)	123.0 ± 14.9	123.5 ± 14.9	124.0 ± 15.0	124.8 ± 15.3	< 0.001
Diastolic BP (mmHg)	76.4 ± 9.8	76.8 ± 9.8	77.1 ± 9.9	77.39 ± 10.0	< 0.001
Fasting glucose (mg/dL)	97.7 ± 21.3	98.4 ± 22.2	99.4 ± 23.3	101.0 ± 25.1	< 0.001
TC (mg/dL)	199.1 ± 35.4	199.9 ± 36.0	200.3 ± 36.8	200.2 ± 38.1	< 0.001
Triglycerides (mg/dL)	111.2 (111.1–111.3)	115.3 (115.2–115.4)	119.4 (119.3–119.5)	124.2 (124.1–124.3)	< 0.001
HDL-C (mg/dL)	54.9 ± 19.4	54.8 ± 19.5	54.7 ± 19.8	54.8 ± 20.0	< 0.001
LDL-C (mg/dL)	119.9 ± 41.3	119.7 ± 41.2	119.3 ± 41.8	117.7 ± 41.4	
AST (U/L)	23.6 (23.6–23.6)	24.1 (24.1–24.1)	24.8 (24.8–24.8)	26.2 (26.2–26.2)	< 0.001
ALT (U/L)	20.5 (20.5–20.5)	21.5 (21.4–21.5)	22.5 (22.5–22.6)	24.3 (24.3–24.3)	< 0.001
GGT (U/L)	22.8 (22.7–22.8)	24.5 (24.5–24.5)	26.9 (26.9–26.9)	32.5 (32.4–32.5)	< 0.001
V1 GGT	22.6 (22.5–22.6)	23.9 (23.9–24.0)	26.0 (26.0–26.1)	31.2 (31.2–31.3)	< 0.001
GGT ASV	1.1 (1.1–1.1)	1.2 (1.2–1.2)	1.4 (1.4–1.4)	1.8 (1.8–1.8)	< 0.001
Serum Cr (mg/dL)	1.03 ± 1.09	1.04 ± 1.14	1.03 ± 1.11	1.0 ± 0.98	< 0.001
eGFR (ml/min/1.73 m^2^)	84.75 ± 33.98	84.79 ± 34.48	85.04 ± 34.23	85.67 ± 34.78	< 0.001
Hemoglobin (g/dL)	13.78 ± 1.52	13.79 ± 1.53	13.80 ± 1.54	13.76 ± 1.54	< 0.001
**Smoking status (%)**					< 0.001
Never smoker	1,014,322 (66.9)	1,004,102 (66.3)	998,277 (65.9)	992,817 (65.5)	
Ex-smoker	239,924 (15.8)	242,688 (16.0)	242,576 (16.0)	241,740 (16.0)	
Current smoker	261,021 (17.2)	267,312 (17.7)	274,025 (18.1)	280,191 (18.5)	
**Alcohol drinking (%)**					< 0.001
Near abstinence	951,494 (62.8)	934,935 (61.8)	927,867 (61.3)	925,666 (61.1)	
Moderate (< 30 g/day)	563,773 (37.2)	579,167 (38.3)	587,011 (38.8)	589,082 (38.9)	
Regular exercise (%)	320,660 (21.2)	320,790 (21.2)	321,505 (21.2)	318,454 (21.0)	< 0.001
**Comorbidities**					
Diabetes (%)	131,942 (8.7)	148,212 (9.8)	173,789 (11.5)	215,391 (14.2)	< 0.001
Hypertension (%)	456,515 (30.1)	480,865 (31.8)	516,984 (34.1)	575,125 (38.0)	< 0.001
Dyslipidemia (%)	329,913 (21.8)	351,454 (23.2)	381,574 (25.2)	429,111 (28.3)	< 0.001
NAFLD (%)	124,838 (8.2)	152,702 (10.1)	188,467 (12.4)	248,520 (16.4)	< 0.001
CKD (%)	103,847 (6.9)	110,335 (7.3)	115,300 (7.6)	121,342 (8.0)	< 0.001
Heart disease (%)	38,537 (3.7)	40,459 (3.9)	42,927 (4.2)	49,409 (4.9)	< 0.001
Stroke (%)	18,592 (1.8)	19,577 (1.9)	20,208 (2.0)	22,992 (2.3)	< 0.001
Admission for HF (%)	1 999 (0.1)	2,207 (0.3)	2,756 (0.2)	4,270 (0.3)	< 0.001
Admission for MI (%)	4,993 (0.3)	5,507 (0.4)	6,329 (0.4)	8,371 (0.6)	< 0.001
Income (lowest 20%, %)	309,047 (20.4)	312,803 (20.7)	316,856 (20.9)	327,392 (21.6)	< 0.001
**Year of V1 exam (%)**					< 0.001
2005	464,166 (30.6)	523,184 (34.6)	514,263 (34.0)	465,242 (30.7)	
2006	509,231 (33.6)	496,850 (32.8)	504,983 (33.3)	526,226 (34.7)	
2007	210,013 (13.9)	198,047 (13.1)	203,840 (13.5)	221,691 (14.6)	
2008	210,539 (13.9)	193,789 (12.8)	195,435 (12.9)	207,588 (13.7)	
2009	89,896 (5.9)	77,317 (5.1)	73,837 (4.9)	73,803 (4.9)	
2010	31,422 (2.1)	24,915 (1.7)	22,520 (1.5)	20,198 (1.3)	
GGT variability assessment period (years)	3.6 ± 0.7	3.7 ± 0.6	3.7 ± 0.6	3.7 ± 0.6	< 0.001
**Follow-up period (years)**	6.4 ± 1.2	6.4 ± 1.2	6.4 ± 1.2	6.3 ± 1.2	< 0.001
< 2.0	6,622 (0.4)	6,903 (0.5)	8,068 (0.5)	11,861 (0.8)	
2.0–3.9	11,996 (0.8)	12,899 (0.9)	14,652 (1.0)	19,550 (1.3)	
4.0–5.9	409,458 (27.0)	378,843 (25.0)	384,127 (25.4)	410,183 (27.1)	
≥ 6.0	1,087,191 (71.8)	1,115,457 (73.7)	1,108,031 (73.1)	1,073,154 (70.9)	

ASV, average successive variability; BMI, body mass index; WC, waist circumference; BP, blood pressure; TC, total cholesterol; HDL-C, high-density lipoprotein-cholesterol; LDL-C, low-density lipoprotein-cholesterol; AST, aspartate transaminase; ALT, alanine aminotransferase; GGT, gamma-glutamyl transferase; Cr, creatinine; eGFR, estimated glomerular filtration rate; CKD, chronic kidney disease; HF, heart failure; MI, myocardial infarction.

Q1: 1–1.16 (men), 1–1.15 (women) U/L; Q2: 1.16–1.27 (men), 1.15–1.26 (women) U/L; Q3: 1.27–1.45 (men), 1.26–1.45 (women) U/L; Q4: 1.45–79.47 (men), 1.45–76.59 (women) U/L. Data are presented as means ± standard deviations, geometric means (95% confidence intervals), or numbers (%). One-way analysis of variance and chi-squared tests were used to compare the characteristics of the study subjects at baseline. A post-hoc multiple comparison analysis was performed with Bonferroni correction, and AST, ALT, GGT, triglyceride levels were log-transformed for analysis.

**Figure 2:**
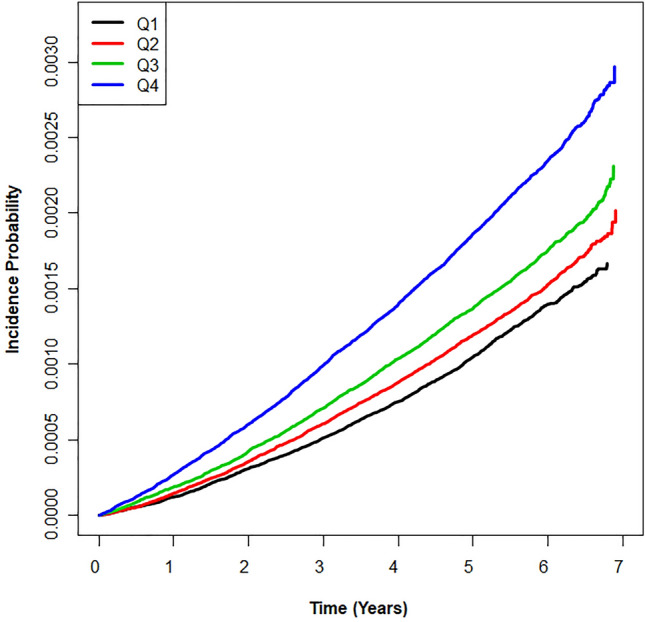
Kaplan–Meier survival curve of outcomes for end-stage renal disease according to quartiles of gamma-glutamyl transferase variability assessed by average successive variability.

**Figure 3:**
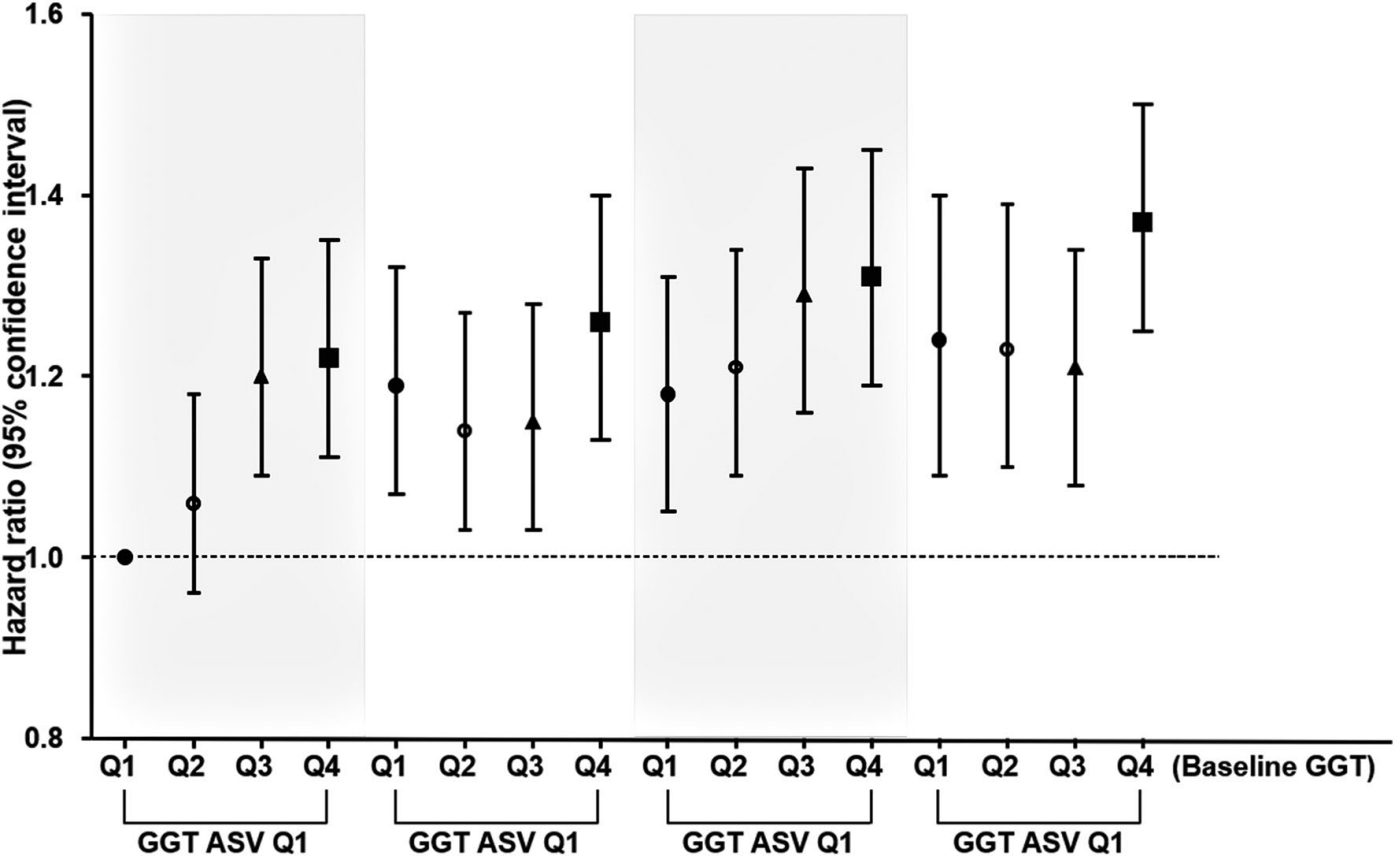
Hazard ratios and 95% confidence intervals for the incidence of end-stage renal disease after dividing the subjects according to quartiles of gamma-glutamyl transferase variability assessed by average successive variability in each quartile of baseline gamma-glutamyl transferase, with adjustment for age, sex, baseline estimated glomerular filtration rate, body mass index, moderate drinking, current smoking, regular exercise, hemoglobin level, and presence of diabetes, hypertension, and dyslipidemia. GGT, gamma-glutamyl transferase; ASV, average successive variability.

During 38,663,279.3 person-years of follow-up, 12,057 of the 6,058,995 subjects (0.2% of subjects) developed ESRD. According to the baseline GGT quartile, the risk for ESRD was significantly higher in GGT quartile 4 than in quartile 1 after adjusting for age, sex, eGFR, and BMI (Table 2). However, the baseline GGT quartile showed a negative association with the risk of ESRD in model 2. In the fully adjusted model 3, the positive relationship was re-observed. We attribute this change to the adjustment for hemoglobin. On the other hand, as the GGT ASV quartile increased, the HR for ESRD increased serially in all three models (Table 2 and Fig. 2). The fully adjusted HRs (95% CIs) for GGT ASV quartiles 3 and 4 were 1.06 (1.01–1.12) and 1.12 (1.06–1.18), respectively. This significance was maintained regardless of baseline GGT quartile (Fig. 3).

**Table 2:** Hazard ratios and 95% confidence intervals for the incidence of end-stage renal disease by quartiles of baseline gamma-glutamyl transferase and average successive variability of gamma-glutamyl transferase.

	Events (n)	Follow-up duration (person-years)	Incidence rate (per 1,000 person-years)	Model 1	Model 2	Model 3
**Baseline GGT quartiles**^a^						
Q1 (n = 1,593,642)	3,254	10,166,664.8	0.32	1(Ref.)	1(Ref.)	1 (Ref.)
Q2 (n = 1,429,570)	2,726	9,141,060.0	0.30	0.95 (0.90–1.00)	0.86 (0.82–0.91)	1.06 (1.00–1.11)
Q3 (n = 1,519,057)	3,029	9,716,606.0	0.31	0.99 (0.94–1.04)	0.81 (0.77–0.85)	1.11 (1.06–1.17)
Q4 (n = 1,516,726)	3,048	9,638,948.5	0.32	1.08 (1.03–1.14)	0.76 (0.72–0.80)	1.15 (1.09–1.21)
*P* for trend				< 0.001	< 0.001	< 0.001
**GGT ASV quartiles**^b^						
Q1 (n = 1,515,267)	2,405	9,645,912.3	0.25	1(Ref.)	1(Ref.)	1 (Ref.)
Q2 (n = 1,514,102)	2,649	9,732,995.2	0.27	1.08 (1.02–1.14)	1.03 (0.97–1.09)	1.01 (0.95–1.07)
Q3 (n = 1,514,878)	3,043	9,705,782.5	0.31	1.23 (1.17–1.30)	1.09 (1.04–1.15)	1.06 (1.01–1.12)
Q4 (n = 1,514,748)	3,960	9,578,589.3	0.41	1.59 (1.52–1.68)	1.27 (1.21–1.34)	1.12 (1.06–1.18)
*P* for trend				< 0.001	< 0.001	< 0.001

GGT, gamma-glutamyl transferase; ASV, average successive variability.

^a^Q1: 4–22 (men), 4–14 (women) U/L; Q2: 23–31(men), 15–18 (women) U/L; Q3: 32–50 (men), 19–25 (women) U/L; Q4: 51–1,000 (men), 26–1,000 (women) U/L.

^b^Q1: 1–1.16 (men), 1–1.15 (women) U/L; Q2: 1.16–1.27 (men), 1.15–1.26 (women) U/L; Q3: 1.27–1.45 (men), 1.26–1.45 (women) U/L; Q4: 1.45–79.47 (men), 1.45–76.59 (women) U/L. Model 1 is adjusted for age, sex, baseline estimated glomerular filtration rate and body mass index. Model 2 is the same as model 1 plus adjustments for moderate drinking, current smoking, regular exercise, and presence of diabetes, hypertension, and dyslipidemia. Model 3 is the same as model 2 plus adjustments for hemoglobin and an income in the lowest 20%. Additionally, baseline GGT was adjusted in the GGT ASV quartiles.

**Figure 4:**
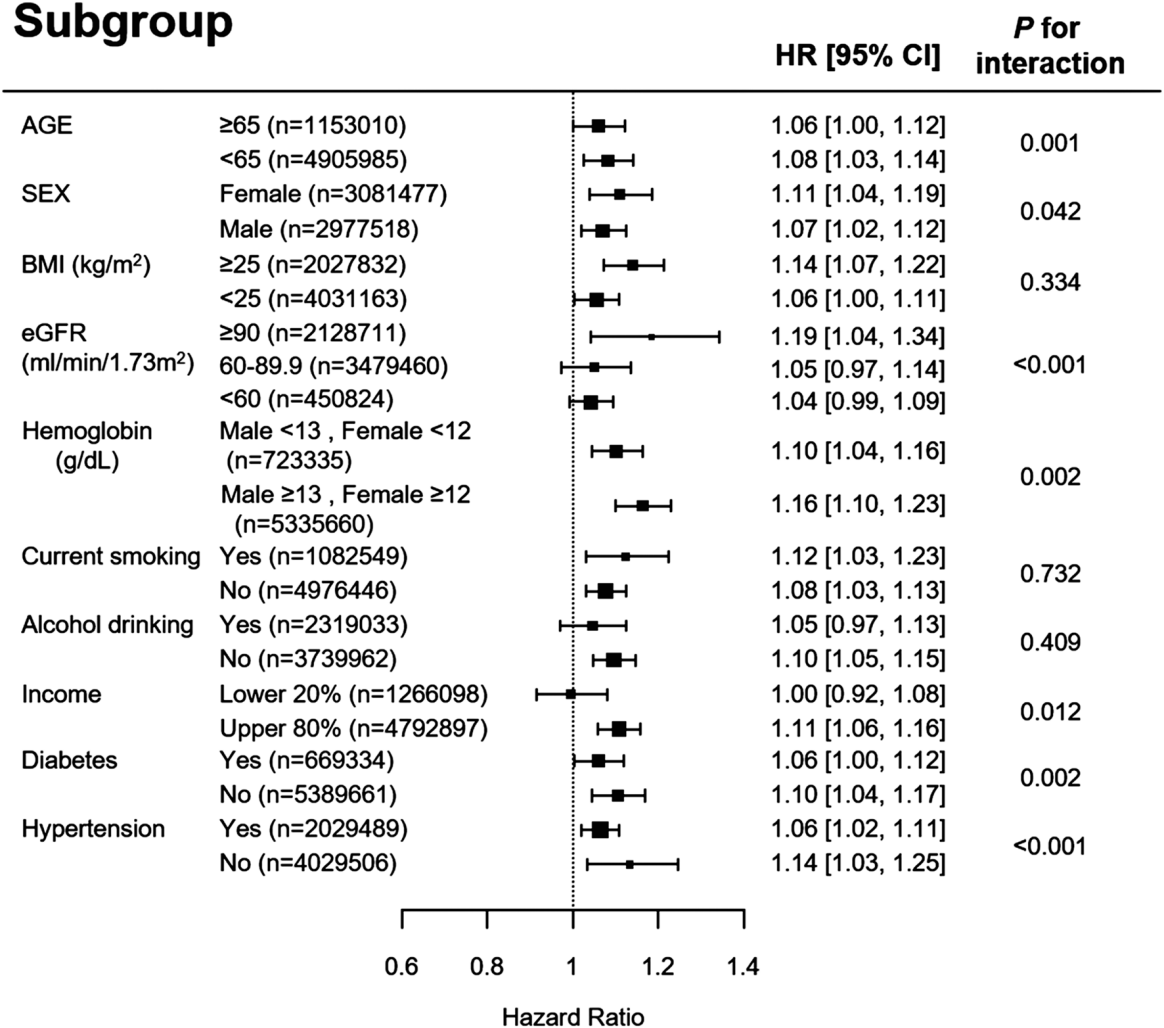
Hazard ratios and 95% confidence intervals for the incidence of end-stage renal disease in the highest quartile versus the other three quartiles of gamma-glutamyl transferase variability assessed by average successive variability in subgroup analyses, with adjustment for age; sex; baseline estimated glomerular filtration rate; body mass index; moderate drinking; current smoking; regular exercise; presence of diabetes, hypertension, and dyslipidemia; hemoglobin; income in the lowest 20%; and baseline gamma-glutamyl transferase level.

In the subgroup analyses, GGT ASV quartile 4 showed an increased risk for ESRD in most of the analyses, with the exceptions being those with baseline eGFR < 90 mL/min/1.73 m^2^, alcohol drinking, and low income (Fig. 4). When we assessed the variability of GGT using SD and CV or excluded ESRD cases within a year (sensitivity analysis), we found similar significances (Supplementary Tables S2 and S3).

## Discussion

In this large population-based study, we demonstrated that GGT variability consistently predicted incident ESRD, independent of various confounders. However, the effects of a single measurement of GGT on the development of ESRD were vulnerable to the adjusted variables.

To date, GGT has been regarded as a marker of metabolic syndrome, increased oxidative stress, and alcohol-related liver disease^14,30^. A significant association between GGT and CKD has also been found^6,9,10^. In studies of a Korean occupational cohort and an urban Han Chinese cohort, increased GGT was associated with an increased risk of future CKD after adjusting for clinically relevant factors^6,9^. However, that significance was confined to men. In a Caucasian male cohort in Finland, the initial evidence disappeared after adjustment for BMI, lipid levels, and lifestyle factors^10^. A population-based cohort study of 185,341 subjects in Austria found that individuals with elevated GGT had an increased relative risk for ESRD during 17.5 years of follow-up^5, 11^. The implications of GGT levels were statistically significant, especially in women and in ESRD caused by diabetic nephropathy^10^.

Few data are available to date to explain the mechanism linking GGT and ESRD risk. Instead, we estimate it through the plausible cause of CKD. First, it can be explained by mechanisms related to oxidative stress^31^. Paradoxically, GGT itself can be a source of reactive oxygen species in the presence of iron^14,31^, which can cause vasoconstriction of the renal vasculature, which results in salt retention and subsequent kidney damage^32^. Second, non-alcoholic fatty liver disease, which has a strong relationship with GGT, could mediate the association between GGT and CKD via hyperglycemia, dyslipidemia, and pro-inflammatory factors^31,33^. Indeed, supporting data show that GGT in arterial atheromatous plaques can promote the further development of atherosclerotic plaques through low-density lipoprotein oxidation^34^.

Given that most previous studies were based on single measurements of GGT, considerable heterogeneity was noted in the thresholds for the GGT levels, ranging from 25 to 61 IU/L^5–7,9–11,30^. In the present study, the baseline GGT levels in quartile 4 were > 50 IU/L for men and > 25 IU/L for women, similar to the definitions of elevated GGT levels used in the previous studies^5–7,9–11,30^. In the present study, however, the risk for ESRD decreased in model 2 as the baseline GGT quartile increased, although a positive relationship was found in models 1 and 3. We observed that hemoglobin levels contributed to that phenomenon. Lower hemoglobin levels in baseline GGT quartile 1 (Supplementary Table S1) might be responsible for the higher risk for ESRD; however, that impact weakened with adjustment for hemoglobin, leading to a lower HR for GGT quartile 1 and higher HR for quartile 4^35^.

In contrast, a significantly increased risk for ESRD in GGT ASV quartile 4 was consistent, irrespective of the baseline GGT level and other confounding factors. Similarly, in most of the subgroup analyses, this significance was observed. Kunutsor et al*.* also previously noted that a single GGT measurement could underestimate the risk of CKD by about 40% compared with analysis using repeated measurements of GGT at intervals of 4 and 11 years^10^.

To our knowledge, this is the first study to investigate the relationship between GGT variability and the development of ESRD in a large general population. Because the serum GGT level is already a component of liver function tests routinely used with low cost, we expect that serial GGT measurements will be a meaningful predictor of ESRD risk^36^.

However, there are limitations to consider. First, we have not specified the main causes of ESRD, which could help to identify people who are particularly vulnerable to the GGT variability. Second, because this study is an observational study, reverse causality is a major concern. Indeed, individuals who were diagnosed with ESRD had elevated GGT ASV compared with the non-ESRD group. To minimize that concern, we conducted a sensitivity analysis after excluding incident ESRD that occurred within 1 year after study inclusion and found similar results. Moreover, consistent findings were observed in subjects whose baseline eGFR was ≥ 60 mL/min/1.73 m^2^. Third, no standard definition of GGT variability has been confirmed. We also computed the SD and CV of GGT as alternative markers for variability and found similar significances in those multivariable-adjusted Cox analyses (Supplementary Table S2). Among those parameters for variability, we have presented the ASV for simplicity. Fourth, we have no information about the subfractions of GGT, which occurs in four types (big, medium, small, and free)^37^. However, although correlation with CVD risk factors differed according to the subfractions of GGT in the Framingham Heart Study cohort^38^, no consensus has been reached about the specific role of each subfraction, and its measurement is not suitable for epidemiologic study. Finally, because some researchers have suggested the possibility that ethnic differences might mediate the effect of GGT^9,10^, the predictive effect of GGT variability should be assessed in different ethnicities.

In conclusion, GGT variability is a novel putative risk factor for ESRD in Asians. We propose serial measurements of GGT to predict ESRD more efficiently.

## Supplementary information

Supplementary Information.

## Data Availability

The data that support the findings of this study are available from the National Health Insurance Corporation but restrictions apply to the availability of these data, which were used under license for the current study, and so are not publicly available. Data are however available from the authors upon reasonable request and with permission of the National Health Insurance Corporation.
