# Supplementary material for: Gamma-glutamyl transferase variability can predict the development of end-stage of renal disease: a nationwide population-based study
Source: Sci Rep. 2020 Jul 15;10:11668. doi: 10.1038/s41598-020-68603-0 (PMC7363906; doi:10.1038/s41598-020-68603-0)
Supplement: Supplementary file 1 — Supplementary Information. [file 41598_2020_68603_MOESM1_ESM.pdf]

Gamma-Glutamyl Transferase Variability can Predict the Development of End-Stage of Renal Disease: A Nationwide Population-Based Study

Running Title: Gamma-Glutamyl Transferase Variability and End-Stage of Renal Disease

Da Young Lee[1], Kyungdo Han[2], Ji Hee Yu[1], Sanghyun Park[2], Ji A Seo[1], Nam Hoon Kim[1], Hye Jin Yoo[1], Sin Gon Kim[1], Seon Mee Kim[3], Kyung Mook Choi[1], Sei Hyun Baik[1], Yong Gyu Park[2] & Nan Hee Kim[1]

[1]Division of Endocrinology and Metabolism, Department of Internal Medicine, Korea University College of Medicine, Seoul, Republic of Korea. [2]Department of Biostatics, College of Medicine, The Catholic University of Korea, Seoul, Republic of Korea. [3]Department of Family Medicine, Korea University College of Medicine, Seoul, Republic of Korea.

Da Young Lee and Kyungdo Han contributed equally to this article.

Correspondence and requests for materials should be addressed to N.H.K. (email: nhkendo@gmail.com) or Y.G.P. (email: ygpark@catholic.ac.kr)

Supplementary Information

Supplementary Table of Contents

Supplementary Figure S1. Flow chart of the study population.

Supplementary Table S1. Baseline characteristics of the study subjects according to quartiles of baseline gamma-glutamyl transferase[a].

Supplementary Table S2. Hazard ratios and 95% confidence intervals for the incidence of end-stage renal disease by quartiles of gamma-glutamyl transferase variability assessed by standard deviation (SD) and coefficient of variation (CV).

Supplementary Table S3. Hazard ratios and 95% confidence intervals for the incidence of end-stage renal disease by quartiles of gamma-glutamyl transferase variability assessed by average successive variability after excluding subjects who developed end-stage renal disease within one year.

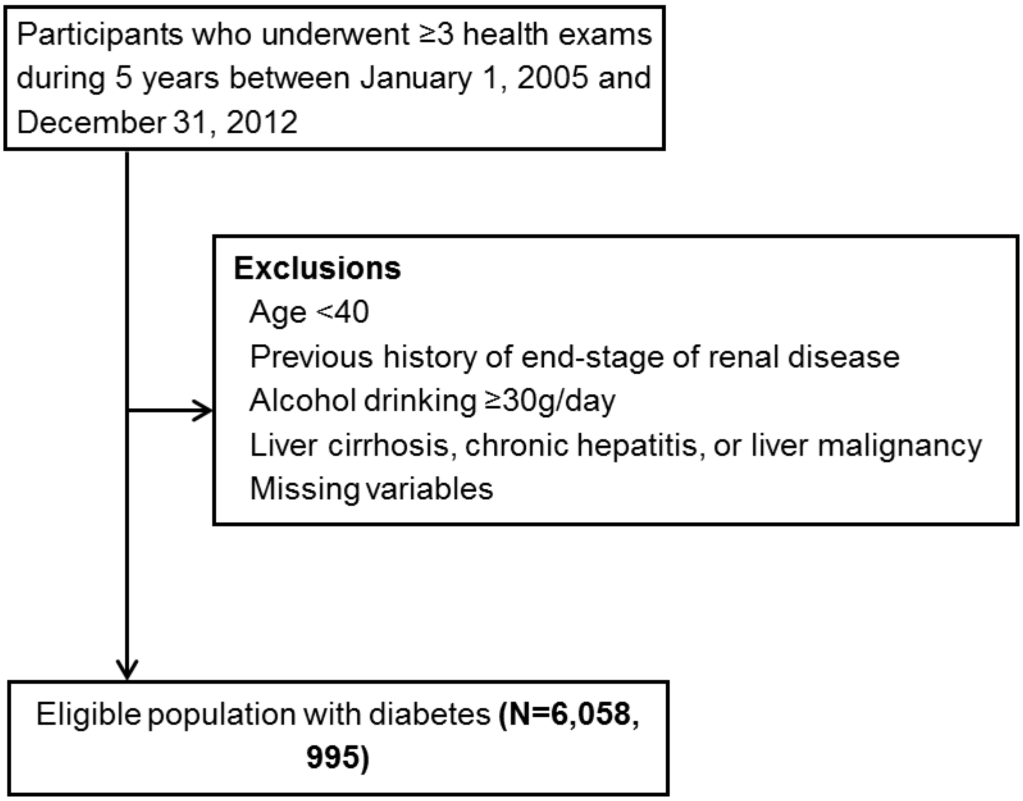

Supplementary Figure S1. Flow chart of the study population.

Supplementary Table S1. Baseline characteristics of the study subjects according to quartiles of baseline gamma-glutamyl transferase[a].

Characteristics	**GGT Q1 (n =1,593,642)**	**GGT Q2 (n =1,429,570)**	**GGT Q3 (n =1,519,057)**	**GGT Q4 (n = 1,516,726)**	***P* value**
Age (years)	55.2 ± 10.9	55.5 ± 10.4	55.6 ± 10.1	55.3 ± 9.7	<0.001
40-64	1,269,613 (79.7)	1,148,734 (80.4)	1,229,171 (80.9)	1,258,467 (83.0)	
≥ 65	324,029 (20.3)	280,836 (19.6)	289,886 (19.1)	258,259 (17.0)	
Sex, male (%)	760,705 (47.7)	700,146 (49.0)	773,548 (50.9)	743,119 (49.0)	<0.001
BMI (kg/m^2)	22.9 ± 2.6	23.6 ± 2.8	24.3 ± 2.9	24.9 ± 3.1	<0.001
WC (cm)	78.1 ± 7.9	80.2 ± 8.2	82.1 ± 8.3	83.7 ± 8.4	<0.001
Systolic BP (mmHg)	120.8 ± 14.7	122.9 ± 14.8	124.9 ± 14.8	126.9 ± 15.1	<0.001
Diastolic BP (mmHg)	74.9 ± 9.7	76.3 ± 9.7	77.6 ± 9.8	79.0 ± 10.0	<0.001
Fasting glucose (mg/dL)	94.9 ± 18.2	97.2 ± 20.5	100.0 ± 23.4	104.6 ± 27.9	<0.001
TC (mg/dL)	190.4 ± 33.4	198.3 ± 34.8	203.2 ± 36.5	207.9 ± 39.2	<0.001
Triglycerides (mg/dL)	94.3 (94.2–94.3)	109.3 (109.2–109.4)	125.7 (125.6–125.8)	147.9 (147.7–148.0)	<0.001
HDL-C (mg/dL)	55.4 ± 19.2	54.9 ± 19.6	54.2 ± 20.2	54.6 ± 19.5	<0.001
LDL-C (mg/dL)	114.9 ± 38.4	119.9 ± 41.1	121.6 ± 41.7	120.4 ± 44.1	
AST (U/L)	22.1 (22.0–22.1)	23.2 (23.2–23.2)	24.7 (24.8–24.7)	29.3 (29.3–29.3)	<0.001
ALT (U/L)	17.4 (17.4–17.4)	19.9 (19.9–19.9)	23.1 (23.1–23.1)	30.4 (30.4–30.4)	<0.001
GGT (U/L)	14.2 (14.2–14.2)	20.8 (20.8–20.8)	29.1 (29.1–29.1)	57.6 (57.5–57.7)	<0.001
V1 GGT (U/L)	16.7 (16.7–16.7)	21.4 (21.4–21.4)	27.8 (27.8–27.8)	44.7 (44.7–44.8)	<0.001
Serum Cr (mg/dL)	1.0 ± 1.1	1.0 ± 1.1	1.0 ± 1.1	1.0 ± 1.1	<0.001
GGT ASV	1.3 (1.3-1.3)	1.3 (1.3-1.3)	1.3 (1.3-1.3)	1.5 (1.5-1.5)	<0.001
eGFR (ml/min/1.73 m^2)	86.3 ± 33.6	84.7 ± 34.6	84.3 ± 34.6	84.9 ± 34.8	<0.001
Hemoglobin (g/dL)	13.5 ± 1.5	13.7 ± 1.5	13.9 ± 1.5	14.1 ± 1.5	<0.001
Smoking status (%)					<0.001
Never smoker	1,128,585 (70.8)	963,158 (67.4)	969,763 (63.8)	948,012 (62.5)	
Ex-smoker	247,730 (15.5)	233,025 (16.3)	255,980 (16.9)	230,193 (15.2)	
Current smoker	217,327 (13.6)	233,387 (16.3)	293,314 (19.3)	338,521 (22.3)	
Alcohol drinking (%)					<0.001
Near abstinence	1,127,120 (70.7)	922,942 (64.6)	892,165 (58.7)	797,735 (52.6)	
Moderate (<30 g/day)	466,522 (29.3)	506,628 (35.4)	626,892 (41.3)	718,991 (47.4)	
Regular exercise (%)	353,531 (22.2)	311,309 (21.8)	319,041 (21.0)	297,528 (19.6)	<0.001
Comorbidities					
Diabetes (%)	112,507 (7.1)	127,063 (8.9)	179,173 (11.8)	250,591 (16.5)	<0.001
Hypertension (%)	390,743 (24.5)	435,224 (30.4)	552,901 (36.4)	650,621 (42.9)	<0.001
Dyslipidemia (%)	234,597 (14.7)	306,303 (21.4)	420,187 (27.7)	530,965 (35.0)	<0.001
CKD (%)	106,069 (6.7)	104,080 (7.3)	119,336 (7.9)	121,339 (8.0)	<0.001
Heart disease (%)	38,417 (3.7)	38,398 (4.0)	45,908 (4.4)	48,609 (4.6)	<0.001
Stroke (%)	21,326 (2.0)	18,846 (2.0)	20,620 (2.0)	20,577 (2.0)	<0.001
Admission for HF (%)	5,361 (0.3)	5,353 (0.4)	6,507 (0.4)	8,135 (0.5)	<0.001
Admission for MI (%)	14,982 (0.9)	14,972 (1.1)	17,224 (1.1)	17,670 (1.2)	<0.001
Income (lower 20%, %)	338,211 (21.2)	297,321 (20.8)	311,635 (20.5)	318,931 (21.0)	<0.001
Year of V1 exam (%)					<0.001
2005	508,961 (31.9)	464,976 (32.5)	499,839 (32.9)	493,079 (32.5)	
2006	542,024 (34.0)	484,363 (33.9)	511,648 (33.7)	499,255 (32.9)	
2007	216,041 (13.6)	195,099 (13.7)	208,753 (13.7)	213,698 (14.1)	
2008	211,209 (13.3)	188,413 (13.2)	199,937 (13.2)	207,792 (13.7)	
2009	86,820 (5.5)	73,624 (5.2)	75,803 (5.0)	78,606 (5.2)	
2010	28,587 (1.8)	23,095 (1.6)	23,077 (1.5)	24,296 (1.6)	
GGT variability assessment period (years)	6.4 ± 1.2	6.4 ± 1.2	6.4 ± 1.2	6.4 ± 1.2	<0.001

Follow-up period (years)				
< 2.0	9,113 (0.6)	7,080 (0.5)	7,804 (0.5)	9,457 (0.6)
2.0–3.9	16,069 (1.0)	13,263 (0.9)	13,954 (0.9)	15,811 (1.0)
4.0–5.9	416,745 (26.2)	369,491 (25.9)	390,542 (25.7)	405,833 (26.8)
≥ 6.0	1 151,715 (72.3)	1 039,736 (72.7)	1 106,757 (72.9)	1 085,625 (71.6)

ASV, average successive variability; BMI, body mass index; WC, waist circumference; BP, blood pressure; TC, total cholesterol; HDL-C, high-density lipoprotein-cholesterol; LDL-C, low-density lipoprotein-cholesterol; AST, aspartate transaminase; ALT, alanine aminotransferase; GGT, gamma-glutamyl transferase; Cr, creatinine; eGFR, estimated glomerular filtration rate; CKD, chronic kidney disease; HF, heart failure; MI, myocardial infarction.

[a]Q1: 4–22 (men), 4–14 (women) U/L; Q2: 23–31(men), 15–18 (women) U/L; Q3: 32–50 (men), 19–25 (women) U/L; Q4: 51–1000 (men), 26–1000 (women) U/L.

Data are presented as means ± standard deviations, geometric means (95% confidence intervals), or numbers (%).
One-way analysis of variance and chi-squared tests were used to compare the characteristics of the study participants at baseline. A post-hoc multiple comparison analysis was performed with Bonferroni correction, and AST, ALT, GGT, triglyceride levels were log-transformed for analysis.

Supplementary Table S2. Hazard ratios and 95% confidence intervals for the incidence of end-stage renal disease by quartiles of gamma-glutamyl transferase variability assessed by standard deviation (SD) and coefficient of variation (CV).

	Events (n)	Follow-up duration (person-years)	Incidence rate (per 1000 person-years)	Model 1	Model 2	Model 3
**GGT SD quartiles [a]**						
Q1 (n=1 514 733)	2,388	9,591,653.7	0.25	1(Ref.)	1(Ref.)	1(Ref.)
Q2 (n=1 514 740)	2,583	9,726,757.4	0.27	1.05 (0.99–1.11)	1.02 (0.96–1.07)	1.00 (0.95–1.06)
Q3 (n=1 514 720)	2,975	9,724,386.4	0.31	1.21 (1.14–1.27)	1.09 (1.03–1.15)	1.05 (0.99–1.11)
Q4 (n=1 514 802)	4,111	9,620,481.8	0.43	1.63 (1.55–1.72)	1.29 (1.23–1.36)	1.13 (1.08–1.20)
P for trend				<0.001	<0.001	<0.001
**GGT CV quartiles [a]**						
Q1 (n=1 514 502)	2,420	9,580,739.5	0.25	1(Ref.)	1(Ref.)	1(Ref.)
Q2 (n=1 515 342)	2,576	9,725,265.0	0.26	1.04 (0.98–1.10)	1.01 (0.96–1.07)	0.99 (0.94–1.05)
Q3 (n=1 514 631)	2,968	9,725,087.4	0.31	1.17 (1.11–1.23)	1.10 (1.04–1.16)	1.04 (0.98–1.10)
Q4 (n=1 514 520)	4,093	9,632,187.4	0.42	1.55 (1.47–1.63)	1.32 (1.26–1.39)	1.10 (1.05–1.16)
P for trend				<0.001	<0.001	<0.001

GGT, gamma-glutamyl transferase; ASV, average successive variability.

Model 1 is adjusted for age, sex, baseline estimated glomerular filtration rate, and body mass index. Model 2 is the same as model 1 plus adjustments for moderate drinking, current smoking, regular exercise, and presence of diabetes, hypertension, and dyslipidemia. Model 3 is the same as model 2 plus adjustments for hemoglobin, an income in the lowest 20%, and baseline GGT.

Supplementary Table S3. Hazard ratios and 95% confidence intervals for the incidence of end-stage renal disease by quartiles of gamma-glutamyl transferase variability assessed by average successive variability after excluding subjects who developed end-stage renal disease within one year.

	Events (n)	Follow-up duration (person-years)	Incidence rate (per 1000 person-years)	Model 1	Model 2
**GGT ASV quartiles**					
Q1 (n=1,513,385)	2,209	8,131,254.8	0.27	1(Ref.)	1(Ref.)
Q2 (n=1,512,028)	2,412	8,219,579.6	0.29	1.07 (1.01–1.14)	1.00 (0.95–1.06)
Q3 (n=1,512,415)	2,741	8,191,726.9	0.33	1.21 (1.14–1.28)	1.03 (0.98–1.09)
Q4 (n=1,511,105)	3,523	8,065,075.3	0.44	1.54 (1.46–1.63)	1.07 (1.01–1.13)
P for trend				<0.001	0.057

GGT, gamma-glutamyl transferase; ASV, average successive variability.

Model 1 is adjusted for age, sex, baseline estimated glomerular filtration rate, and body mass index. Model 2 is the same as model 1 plus adjustments for moderate drinking, current smoking, regular exercise, and presence of diabetes, hypertension, and dyslipidemia. Model 2 is the same as model 1 plus adjustments for moderate drinking; current smoking; regular exercise; presence of diabetes, hypertension, and dyslipidemia; hemoglobin, an income in the lowest 20%, and baseline GGT.
